# Assessment of adherence to dietary recommendations and associated factors among type 2 diabetic patients in selected hospitals in Addis Ababa, Ethiopia

**DOI:** 10.3389/fnut.2024.1474445

**Published:** 2025-02-10

**Authors:** Wabi Temesgen Atinafu, Kefyalew Naniye Tilahun

**Affiliations:** Department of Public Health, College of Medicine and Health Science, Ambo University, Ambo, Ethiopia

**Keywords:** adherence, diabetes mellitus, dietary recommendations, Addis Ababa, Ethiopia

## Abstract

**Background:**

In Ethiopia, diabetes and its complications are significant causes of morbidity and mortality, with huge economic implications. Despite the high dietary non-adherence that has been reported in limited studies in Ethiopia. Moreover, there is a gap from the perspective of patients in the area of this topic.

**Objectives:**

This study aimed to assess adherence to dietary recommendations and associated factors among type 2 diabetic patients in Addis Ababa Selected Hospitals, Addis Ababa, Ethiopia, 2024.

**Methods and materials:**

An institution-based cross-sectional study was conducted in a 420 sampled population among four hospitals found in Addis Ababa, Ethiopia, from 24 June to 15 July 2024. Systematic random sampling was used to recruit individual study participants. The collected data were exported into SPSS version 25 software for analysis. A descriptive summary, including frequencies, percentages, and graphs, was used to present the study results. Binary logistic regression was used for statistical analysis. Finally, the results of bivariate and multivariable logistic regression analyses were presented using odds ratios with 95% confidence intervals. In the final model, a *p*-value of <0.05 was taken as statistically significant.

**Results:**

A total of 406 participants were included, with a response rate of 96.7%. The study participants had an average age of 48 (± 11.4 years), and the overall adherence to dietary recommendations in this study was 62.8% among type 2 diabetic patients. Years of diagnosis of DM, having a family history of DM, having comorbidities, and having received diabetic nutrition education were significantly associated with adhering to diabetic dietary recommendations among type 2 diabetic patients.

**Conclusion and recommendation:**

The study found that overall adherence to dietary recommendations among type 2 diabetic patients was relatively high at 62.8%, suggesting the need to develop and implement tailored dietary education and counseling programs for this patient population.

## Introduction

Diabetes is a chronic metabolic disorder defined by elevated blood glucose (or blood sugar) levels that leads to catastrophic damage to the heart, blood vessels, eyes, kidneys, and nerves over time, according to the World Health Organization (1). Diabetes affects approximately 422 million people globally, the majority of whom live in low- and middle-income countries, and diabetes is directly responsible for 1.6 million fatalities per year. Over the last few decades, both the number of cases and the prevalence of diabetes have significantly increased (1). By 2025, a global agreement has been reached to halt the rise in diabetes and obesity (2, 3).

The significant increase in overweight, obesity, and physical inactivity rates may be partly responsible for this global epidemic (2). The most prevalent type of diabetes, type 2 diabetes (accounting for 90% of cases), affects adults and occurs when the body becomes resistant to insulin or does not produce enough of it (4).

Ethiopia is one of the top five countries in sub-Saharan Africa with the highest number of individuals affected by diabetes (5–7). Diabetes and its complications are the leading causes of illness and mortality in Ethiopia, with severe economic effects (6). The prevalence of diabetes is rapidly growing in Ethiopia. According to reports, the prevalence of diabetes in Ethiopia ranged from 2 to 6.5%, with the lowest 2% in rural areas (5). Oral hypoglycemia and insulin were the most common treatments (8). Retinopathy (2.7–25%), nerve damage (4.8–35.0%), depression (13–61.0%), kidney illness (18.2–23.8%), hypertension (23–54.82%), anemia (19%), and related costs were the most commonly reported diabetes-related problems in the country (5, 6, 8).

Dietary practice refers to patients’ food preferences based on diabetes education, emphasizing the consumption of foods containing less fat, more fiber, and less sodium (9). Daily carbohydrate, protein, and fiber intake should be 45–50%, 10–20%, and 12% of total calories, with a minimum of 0.5 g of fat per meal (10). Nutrition is crucial for diabetes management and prevention.

The risk of T2DM is connected to both malnutrition and overnutrition (11). Balanced food intake with endogenous and/or exogenous insulin levels is the most important for diabetes treatment in improving glycemic control (12). However, for many DM patients, deciding what to eat and adhering to the meal plan are the most difficult aspects of the treatment strategy. There is no one-size-fits-all eating pattern for those with diabetes (13).

According to a study conducted at Gondar Comprehensive Specialized Hospital in Ethiopia, 48.3% of participants had poor adherence to dietary recommendations. and a study conducted in northern Ethiopia found that 74.3% of participants also had poor adherence to dietary recommendations. In Gondar Comprehensive Specialized Hospital and study conducted in general Ethiopian hospitals, respectively (14, 15). Another study conducted in Ethiopian teaching hospitals showed that the overall adherence to dietary recommendations was 44.3% among diabetic patients who participated in the study (16). Furthermore, study findings in Jimma, Ethiopia, have shown that the level of diabetic self-care practice was insufficient among the study participants (17).

In developed countries, dietary compliance among people with type 2 diabetes has improved. In affluent countries, dietary adherence among type 2 diabetes patients has improved and is currently considered satisfactory. However, in underdeveloped nations, dietary adherence among type 2 diabetes patients is given minimal attention and is not regarded as a major national priority. Diabetes has improved and is now relatively satisfactory.

In developing countries, there is minimal focus on dietary adherence for patients with type 2 diabetes, and it is not given priority at the national level. Due to the importance of involving patients in chronic disease management, this study aims to show the level of adherence to dietary recommendations and identify associated factors among type 2 DM patients in selected hospitals in Addis Ababa, Ethiopia, in 2024.

## Materials and methods

### Study design and period

An institutional cross-sectional study was conducted from 24 June to 15 July 2024 among type 2 DM patients in selected hospitals in Addis Ababa, Ethiopia, in 2024.

### Study participants and sample size determination

The population for this study consisted of all adult type 2 diabetic patients visiting non-communicable disease clinics in selected hospitals in Addis Ababa, Ethiopia. Patients who attended these hospitals during the study period were considered the study population. All type 2 diabetes patients who had at least one follow-up visit at the selected hospitals after diagnosis before the data collection period were included in the study, and newly diagnosed type 2 diabetes of less than 1 month, pregnant and lactating women, and those requiring constant medical support and monitoring were excluded from this study.

The sample size was obtained using a single population proportion formula: $n=\frac{{z_{\alpha /2}}^{2}\;P\left(1-P\right)}{D^{2.}}$

where *n* = sample size, $z_{\alpha /2}$ = level of confidence 95%, or reliability coefficient = 1.96, P = proportion of the population = 53.7%, poor dietary practice among type 2 diabetes mellitus patients in Nigist Eleni Mohammed Memorial Teaching Hospital (18), and D = margin of error (0.05). Thus, the final sample size for this study was 420 type 2 diabetes patients.

### Sampling procedures

The study was conducted in 4 randomly selected hospitals out of 13 government hospitals in Addis Ababa, Ethiopia. A systematic random sampling technique was used to select the study participants. The study participants were chosen from the selected hospitals in Addis Ababa, Ethiopia. The total number of type 2 DM patients who visited the selected hospitals during the data collection period was calculated based on the hospitals’ previous monthly reports. The calculated sample size was then distributed proportionally across each hospital according to the number of type 2 DM patients at each location. The first respondent was selected randomly.

### Data collection tools and procedure

Data were collected using a pretested structured questionnaire administered via face-to-face interview. The questionnaire was adapted from various previously published studies (14, 16, 18, 19). The questions were initially prepared in English, then translated into Amharic, and then back-translated to English by language experts to ensure consistency. Four data collectors (BSC nurses) who had prior experience in data collection collected the data, and one experienced public health professional was assigned as supervisor. One-day training was given to the data collectors and supervisors for the data collection process. The list of selected participants to be interviewed was located in the follow-up waiting rooms of the hospitals. The completeness, consistency, and accuracy of the collected data were examined every day.

Adherence to dietary recommendations, the dependent variable in this study, was defined as adherent when the participant’s score on the item used to measure the dietary adherence assessment scale was equal to or above the mean score and non-adherent when the score was below the mean scale (16).

### Data quality control

To ensure data quality, supervisors and data collectors were given one-day training on the purpose of the study, data collection tools, and procedures. The questionnaire was translated from English to Amharic, and to check for the original meaning, the Amharic version was re-translated to English. The questionnaire was pre 5% of the calculated sample size out of the study area before 2 weeks of the main data collection. The collected data were checked by reviewing all the questionnaires to ensure completeness and consistency of the information collected.

### Data processing and analysis

The collected data were visually checked by the investigator, and then the data were coded and entered into Epi Data version 3.1 software before being exported into SPSS version 25 software for further analysis. Data cleaning was conducted before the analysis. Mean and standard deviation were calculated for continuous variables. A descriptive summary using frequencies, percentages, and graphs was used to present the study results. Then, logistic regression analysis was applied to assess the association between the dependent and explanatory variables. The degree of association was interpreted using odds ratios with 95% confidence intervals, and *p*-values less than 0.05 were considered statistically significant. Bivariate and multivariate analyses were used to identify characteristics that influence the recommended dietary adherence. The variance inflation factor (VIF) was used to test the multicollinearity assumption, and the results ranged from 1 to 10, suggesting no collinearity. Cross-tabulation was used for each independent variable to better understand cell information and to correct for extreme cell values in the logistic regression analysis. The model’s fitness was evaluated using the Hosmer–Lemeshow goodness-of-fit test, which yielded a *p*-value of 0.76, suggesting that the model was well-fitted.

## Results

### Socio-demographic characteristics of respondents

The study planned to include 420 participants, and 406 patients volunteered to participate, resulting in a response rate of 96.7%. Approximately half of the study participants were men (220, 54.2%), while the other half were women (186, 45.8%). The study participants had an average age of 48 (± 11.4 years), with the majority, 168 participants (41.4%), being above >60 years old. The educational status of participants revealed that 38.9% of the study participants had attended college or higher education (Table 1).

**Table 1:** Socio-demographic characteristics of study participants among type 2 diabetic patients in selected hospitals, in Addis Ababa, Ethiopia, 2024.

Variables	Categories	Frequency	Percent
Age in years	<20 20–40 41–60 >60	38 77 123 168	9.3% 19% 30.3% 41.4%
Sex	Male Female	220 186	54.2% 45.8%
Marital status	Single Married Divorced Widowed	17 329 34 26	4.4% 81% 8.2% 6.4%
Religion	Orthodox Protestant Muslim Others^1^	164 142 71 29	40.4% 35% 17.5% 7.1%
Occupation	Government employee Private employee Merchant Others	112 117 108 69	27.6% 28.8% 26.6% 17%
Educational status	No formal learning Primary school Secondary school College and above	46 85 117 158	11.4% 20.9% 28.8% 38.9%
Household monthly income	<4,000 4,000–8,000 >8,000	115 204 87	28.3% 50.2% 21.5%

1 Others, Catholic Judith, Waqefata, Adventist.

### Health-related characteristics of respondents

Table 2 illustrates the distribution of health-related characteristics of the study participants. The participants who had the condition for greater than 5 years were 219 (53.9%), and 187 of the participants were less than or equal to 5 years ago after they were diagnosed with the disease. The majority of individuals, (349, 86%) were on oral hypoglycemic medication, with 57 (14%) using both oral medication and insulin. Among the study participants, 182 (44.8%) had comorbidities, and the majority, (310, 76.4%), had no family history of diabetes. Among the study participants, 254 (62.5%) patients had a BMI between 18.5 and 24.5 kg/m2, 26 (6.3%) patients had a BMI less than 18.5 kg/m2, and 66 (16.3%) of the study participants had a BMI between 24.5 and 29.5 kg/m2 (Table 2).

**Table 2:** Health-related characteristics of the study participants among type 2 diabetic patients in selected hospitals, Addis Ababa, Ethiopia, 2024.

Variables	Categories	Frequency	Percent
Years of diagnosis for Diabetes	≤5 >5	187 219	46.1% 53.9%
Comorbid conditions	Yes No	182 224	44.8% 55.2%
Family history of DM	Yes No	96 310	23.6% 76.4%
Encountered any of the complications of DM	Yes No	128 278	31.5% 68.5%
Complications of DM encountered	Diabetic foot ulcer Renal diseases Eye condition Diabetic neuropathy Amputation	23 16 33 44 12	18% 12.5% 25.8% 34.4% 9.3%
Treatment medication for DM	Oral hypoglycemic drugs Both oral hypoglycemic drugs and insulin	349 57	86% 14%
Diabetes nutritional education	Yes No	316 90	77.8% 22.2%
BMI kg/m^2^.	<18.5 kg/m2. 18.5–24.5 kg/m2. 24.5–29.5 kg/m2. >30 kg/m2.	26 254 66 60	6.4% 62.5% 16.3% 14.8%

### Behavioral-related factors

The majority of study participants had never consumed drinks containing alcohol (198, 48.7%). Additionally, approximately 347 participants (85.5%) were not currently smoking cigarettes, and 162 participants (39.9%) engaged in physical exercise at least once per week, as much as they were able (Table 3).

**Table 3:** Behavioral-related characteristics of the study participants among type 2 diabetic patients in selected hospitals, Addis Ababa, Ethiopia, 2024.

Variables	Categories	Frequency	Percent
Frequency of drink containing alcohol	Never <2 per day 2–4 per day >4 per day	198 68 59 81	48.7% 16.8% 14.5% 20%
Currently smoke cigarette	Yes No	59 347	14.5% 85.5%
Doing physical exercise	Yes No	162 244	39.9% 60.1%

### Adherence to dietary recommendation among type 2 diabetic patients in selected hospitals, Addis Ababa, Ethiopia, 2024

The overall adherence to dietary recommendations according to our research findings is 255 (62.8%) (95% CI, 54.3, 67.4) among diabetic patients who participated in the study, and 129 participants (31.8%) were not adherent to the dietary recommendation.

### Factors associated with adherence to the recommended dietary regime among type 2 diabetic patients in selected hospitals, Addis Ababa, Ethiopia, 2024

The following independent variables were candidates for the final model in the bivariate logistic regression analysis: family history of DM, presence of comorbidities, duration of diagnosis, physical activities, educational status, and diabetic nutritional education. In the multivariable logistic regression analysis, family history of DM, presence of comorbidities, duration of diagnosis, and receiving diabetic nutritional education were significantly associated with adherence to dietary recommendations among type 2 diabetic patients.

Our study shows that patients who had a family history of DM were 1.58 times more likely to adhere to dietary recommendations as compared to those patients who had no family history of DM [AOR 1.58 = 95%CI (1.06–2.10)]. The odds of adhering to dietary recommendations among type 2 diabetic patients were 1.08 times greater in patients who had comorbidities compared to those who had no history of comorbidity conditions [AOR = 1.08, 95%CI (1.02–1.14)].

Our study findings show that patients who were diagnosed with type 2 diabetic cases before 5 years were 1.46 times more likely to adhere to dietary recommendations as compared to those patients who were diagnosed with type 2 diabetic cases less than 5 years [AOR = 1.46, 95%CI (1.02–1.90)]. Additionally, patients who received diabetic nutrition education were 1.14 times more likely to adhere to the recommended dietary guidelines than those who did not receive nutritional education [AOR = 1.14, 95%CI (1.04–95)] (Table 4).

**Table 4:** Bivariate and multiple logistic regression analyses of factors associated with adherence to the recommended dietary regime among type 2 diabetic patients in selected hospitals, Addis Ababa, Ethiopia, 2024.

Variables	Categories	Adherence		COR (95% CI)	AOR (95% CI)	*p*-value
		Yes	No			
Family history of DM	No	203	107	1	1	
	Yes	52	44	1.61(1.07–2.15)	1.58(1.06–2.10)	0.001*
Presences of comorbidities	No	143	81	1		
	Yes	112	70	1.10(1.02–1.18)	1.08(1.02–1.14)	0.004*
Duration of the diagnosis	<=5	126	61	1	1	
	>5	129	90	1.44(1.08–1.80)	1.46(1.02–1.90)	0.001*
Physical activities	Yes	118	44	1	1	
	No	137	107	2.09(1.23–2.95)	2.07(0.87–3.27)	1.24
Educational status	No formal education	24	22	1	1	
	Primary school	48	37	0.84(0.22–1.46)	0.84(0.22–1.46)	0.87
	Secondary school	77	40	0.67(0.46–1.06)	0.64(0.25–1.03)	0.79
	College and above	106	52	0.94(0.91–0.97)	0.92(0.78–1.06)	0.91
Diabetic nutritional education	No	59	31	1	1	
	Yes	196	120	1.17(1.03–1.97)	1.14(1.04–95)	0.003*

*Statistically significant.

## Discussion

The purpose of this hospital-based cross-sectional study was to measure the proportion of adherence to dietary recommendations and associated factors among type 2 diabetic patients in selected hospitals, in Addis Ababa, Ethiopia, in 2024. The findings indicate that the overall adherence to the dietary recommendation in this study was 255 (62.8%) (95% CI, 54.3, 67.4) among type 2 diabetic patients who participated in the study, and 129 participants (31.8%) were not adherent to the dietary recommendation. Adherence to the appropriate diet for diabetes patients is particularly crucial to achieving optimal metabolic management, as non-adherence is related to increased glucose and cholesterol levels, which eventually lead to serious problems (20). Diabetes and its complications are widely recognized as a major global burden, presenting considerable challenges to patients, healthcare systems, and national economies (21).

According to our results, a total of 62.8% of participants adhered to dietary recommendations. This finding is comparable to studies conducted in Southern Ethiopia (64%) (22), Ghana (65.1%) (23), Kuwait (63.5%) (24), and Saudi Arabia (67.2%) (25). The populations in these regions may share similar dietary patterns, food preferences, and socioeconomic backgrounds that influence their ability to adhere to recommended dietary guidelines. However, this proportion is higher than that found in studies conducted in Dire Adwa (37.5%) (26), Debre Tabor (25.7%) (27), and Bahir Dar (53.2%) (28). This disparity could be attributed to differences in the study demographic, sample size, and adherence measuring technique, as well as the difficulty of defying social expectations or demands to eat with friends and family or the necessity of eating out.

This result is lower than the findings of studies conducted in Surat City (76.2%) (29) and Delhi, India (84.6%) (30). The disparity could be explained by the variation in the study settings, differences in socioeconomic status, and availability of different types of foods in the two countries.

These patients who were diagnosed with type 2 diabetic cases before 5 years were 1.46 times more likely to adhere to dietary recommendations as compared to those patients who were diagnosed with type 2 diabetic cases less than 5 years. This finding is in line with studies conducted in Debre Berhan (16), Addis Ababa (9), Gondar (31), Bahir Dar (28), and Nigeria (32). A possible explanation is that patients with diabetes for a longer period of time have more frequent contact with health professionals and are more likely to receive repetitive instructions on adhering to dietary recommendations. This increased exposure may help them become more aware of the acute and chronic complications of uncontrolled blood glucose, ultimately leading to the adoption of healthier behaviors.

Our findings demonstrate that patients with a family history of diabetes were 1.58 times more likely to follow dietary recommendations than those who did not have a family history. This finding is similar to studies conducted in Gondar (31), Eastern Ethiopia (26), and Southwest Ethiopia (22). This might be because having a family member with diabetes could be a good source of information about the disease process and how to control blood glucose.

The odds of adhering to dietary recommendations among type 2 diabetic patients were 1.08 times greater in patients who had comorbidities compared to those who had no history of comorbidity conditions. This finding is in line with studies conducted in Debre Tabor (27) and Gondar (31). Patients with comorbidity may be on complex medication regimens and dietary advice. As a result, providing comprehensive information on the benefits of dietary adherence, particularly for diabetic patients with comorbidities, is a crucial strategy to improve overall health outcomes.

Patients who received diabetic nutrition education were 1.14 times more likely to adhere to the suggested diet for their conditions than patients who did not receive nutritional education.

This finding aligns with studies conducted in Addis Ababa (9) and Debre Berhan (16). This may be due to the fact that those who received nutrition education are more likely to follow the advice of clinicians and have a better understanding of the food-disease association, food guides, and dietary prescriptions than those who did not receive nutrition education. In addition, patients who have received nutrition education may perceive a greater level of seriousness of not adhering to the recommended dietary regimen.

### Limitations of the study

This study has limitations that must be considered while interpreting the results. This study may not show temporal relationships of potential risk factors with dietary recommendation adherence due to the cross-sectional nature of the design used. Using self-reported dietary recommendation adherence as a measure of the level of practice may introduce social desirability bias. The dietary recommendation adherence scale has not been validated before, and it is likely that our estimates may underestimate or overestimate the outcome. Moreover, readers and researchers should be aware that the findings of the present study may not represent the population of the entire country as a source of food and other factors may vary across different geographical areas.

## Conclusion

According to our findings, the overall adherence to dietary recommendations in this study was 62.8% among type 2 diabetic patients who participated in the study, which is relatively high. Years of diagnosis of DM, having a family history of DM, having comorbidities, and having received diabetic nutrition education were significantly associated with adhering to diabetic dietary recommendations among type 2 diabetic patients who had follow-ups at selected public hospitals in Addis Ababa, Ethiopia. Healthcare providers can develop effective strategies to improve dietary adherence, such as following dietary plans, providing dietary education, and tailoring dietary interventions to patients’ preferences. Additionally, tailored dietary education and counseling programs should be developed and implemented specifically for type 2 diabetic patients with comorbidities, and diabetic nutrition education should be established as a standard component of comprehensive care plans for type 2 diabetes management.

## Data Availability

The datasets presented in this article are not readily available because some of the datasets contain sensitive information that cannot be shared publicly due to participant confidentiality and data protection regulations. Requests to access the datasets should be directed to the corresponding author.
